# Supplementary material for: A computational approach to aid clinicians in selecting anti-viral drugs for COVID-19 trials
Source: Sci Rep. 2021 Apr 27;11:9047. doi: 10.1038/s41598-021-88153-3 (PMC8079380; doi:10.1038/s41598-021-88153-3)
Supplement: Supplementary file 1 — Supplementary Information. [file 41598_2021_88153_MOESM1_ESM.pdf]

SUPPLEMENTARY:
A computational approach to aid clinicians in selecting anti-viral drugs for COVID-19 trials

Aanchal Mongia[1], Sanjay Kr. Saha[2], Emilie Chouzenoux[3*] & Angshul Majumdar[4*]

[1]Dept. of CSE, IIIT - Delhi, India, 110020
[2]Department of Community Medicine, IPGMER Kolkata
[3]CVN, Inria Saclay, Univ. Paris Saclay, 91190 Gif-sur-Yvette, France
[4]Dept. of ECE, IIIT - Delhi, India, 110020
[*]Corresponding authors contact/Email:
angshul@iiitd.ac.in, emilie.chouzenoux@centralesupelec.fr

August 3, 2020

1 Description of drugs predicted by computational models

- **Remdesivir** (FDA emergency use, investigational) – Remdesivir was originally investigated as a treatment for Ebola virus, but has potential to treat a variety of RNA viruses. Its activity against the coronavirus (CoV) family of viruses, such as SARS-CoV and MERS-CoV, was shown in 2017, and it is also being investigated as a potential treatment for COVID-19.

- **Ribavirin** (approved) – Broad-spectrum activity against several RNA and DNA viruses. It is primarily indicated for use in treating hepatitis C and viral hemorrhagic fevers. It is reported that ribavirin might be only effective in early stages of viral hemorrhagic fevers including Lasser fever, Crimean-Congo hemorrhagic fever, Venezuelan hemorrhagic fever, and Hantavirus infection. Currently Ribavirin is being used in combination with interferon beta-1b, lopinavir–ritonavir in a trial for treating COVID-19.

- **Umifenovir** (Investigational) – Umifenovir is used for the treatment and prophylaxis of influenza and other respiratory infections. Umifenovir's ability to exert antiviral effects through multiple pathways has resulted in considerable investigation into its use for a variety of enveloped and non-enveloped RNA and DNA viruses, including Flavivirus / hemorrhagic fever, Zika virus, Lassa virus, Ebola virus, chikungunya virus, Hantaan virus, and coxsackie virus A and B. Umifenovir is currently being investigated as a potential treatment and prophylactic agent for COVID-19.

- **Sofosbuvir**– Sofosbuvir is a direct acting antiviral medication used as part of combination therapy to treat chronic Hepatitis C. Currently it is undergoing clinical trial for treating COVID-19.

- **Taribavirin** (similar to Ribavirin) – The prodrug taribavirin is under development for the treatment of patients with chronic hepatitis C. Taribavirin is metabolized by the liver and converted into its active metabolite, ribavirin. This pathway reduces exposure to red blood cells (RBCs) and increases exposure to the liver, the site of HCV replication.

- **Tenofovir alafenamide** – Tenofovir alafenamide is a novel tenofovir prodrug developed in order to improve renal safety when compared to the counterpart tenofovir disoproxil. Tenofovir alafenamide is indicated to treat chronic hepatitis B, treat HIV-1 and prevent HIV-1 infections. Currently tenofovir disoproxil is undergoing trial as a prophylactic against COVID-19 on healthcare workers. The study is supposed to be completed in July.

2 Symptoms of different virus infections

- **Hemorrhagic fever symptoms (Mayoclininc, Clevelandclinic)** – fever, fatigue, dizziness, body ache, headache, rashes, bleeding from the eyes, ears or mouth, difficulty breathing, internal bleeding, organ failure.
  Drugs: Ribavarin, Umifenovir

  SARS-CoV symptoms (CDC, WHO) – fever, dry cough, sore throat, shortness of breath, headache, body ache, loss of appetite, malaise, night sweats, chills, confusion, rash and diarrhea.
  Drugs: Remdesivir

  MERS-CoV symptoms (CDC, WHO) – fever, chills, headache, body ache, malaise, shortness of breath, diarrhea, dry cough, sore throat, body ache and hypoxia.
  Drugs: Remdesivir

  Influenza virus (CDC) – fever, body ache, chills, sweats, headache, dry cough, fatigue, nasal congestion and sore throat.
  Drugs: Umifenovir, Ibuprofen

  Zika virus (CDC) - mild fever, rash, body ache, headache, red eyes and malaise.
  Drugs: Umifenovir

  Lassa virus (WHO) – fever, fatigue, malaise, headache, sore throat, body ache, chest pain, nausea, vomiting, diarrhoea, cough and abdominal pain.
  Drugs: Umifenovir, Ribavarin

  Ebola virus (CDC, WebMD) – fever, body ache, headache, abdominal pain, rash, loss of appetite, fatigue, diarrhea, vomiting, unexplained hemorrhaging, bleeding or bruising.
  Drugs: Remdesivir, Umifenovir

  Chikungunya virus (CDC) – fever, body ache, headache, joint swelling and rash.
  Drugs: Umifenovir

  Hantavirus (CDC) – fatigue, fever, body ache, headache, dizziness, chill, nausea, vomiting, diarrhea, abdominal pain, blurred vision, inflamed or red eyes, shortness of breath, rash, low blood pressure. Drugs: Umifenovir, Ribavarin coxsackie virus A (WebMD) – sore throat, blisters in mouth, and small tender lesions on the palms of their hands and bottom of their feet, inflammation of the spinal cord and brain.
  Drugs: Umifenovir

  Coxsackie virus B (WebMD) – fever, spasms of the abdominal and chest muscles, inflammation of the spinal cord and brain.
  Drugs: Umifenovir

  Non-polio Enterovirus (CDC) – fever, runny nose, sneezing, cough, skin rash, mouth blisters, body muscle aches.
  Drugs: Pleconaril

  Rhinovirus (Mayoclinic) – runny nose, sore throat, cough, congestion, mild body aches, mild headache, sneezing, low fever and malaise.
  Drugs: Ribavarin, Sofosbuvir, Taribavirin

  Hepatitis B (DNA virus) (hepb.org) – fever, fatigue, body ache, loss of appetite, nausea, vomiting, stomach pain, pale or light colored stools, dark urine, jaundice, bloated stomach.
  Drugs: Tenofovir alafenamide

  HIV-1 (acute phase) – fever, chills, rash, night sweats, body ache, sore throat, fatigue, swollen lymph nodes, mouth ulcers.
  Drugs: Tenofovir alafenamide

3 Readme for Webserver

The web server (`http://dva.salsa.iiitd.edu.in`) has two tabs in the menu. The 'Home' page provides reference to our work and the main functionalities of the web server. The 'About' page consists of a brief description of the dataset and the algorithms used.

Home – The 'Home' page briefly discusses what this web server is about. It also includes reference to our pre-print. In the sub-section 'Functionalities', there are two parts. The first one pertains to the prediction of drugs, given the genomic structure of the virus. The second takes in two inputs, a drug and a virus, and it returns a normalized score depicting the overall efficacy of the drug against the virus, as predicted by our computational model.

Figure 1 shows the 'Home' page of the server. Figures 2,3,4,5 show how to use the web server to run an algorithm for the prediction of top-5 drugs for a virus already existing in the database (sample query 1: Figures 2 and 3) or for a novel virus by uploading its genomic sequence in ".fasta" file format (sample query 2: Figures 4 and 5). Figures 6 and 7 depict the usage of the software to predict the association score (normalized) between a drug and a virus predicted by the chosen algorithm (sample query 3: Figures 6 and 7).

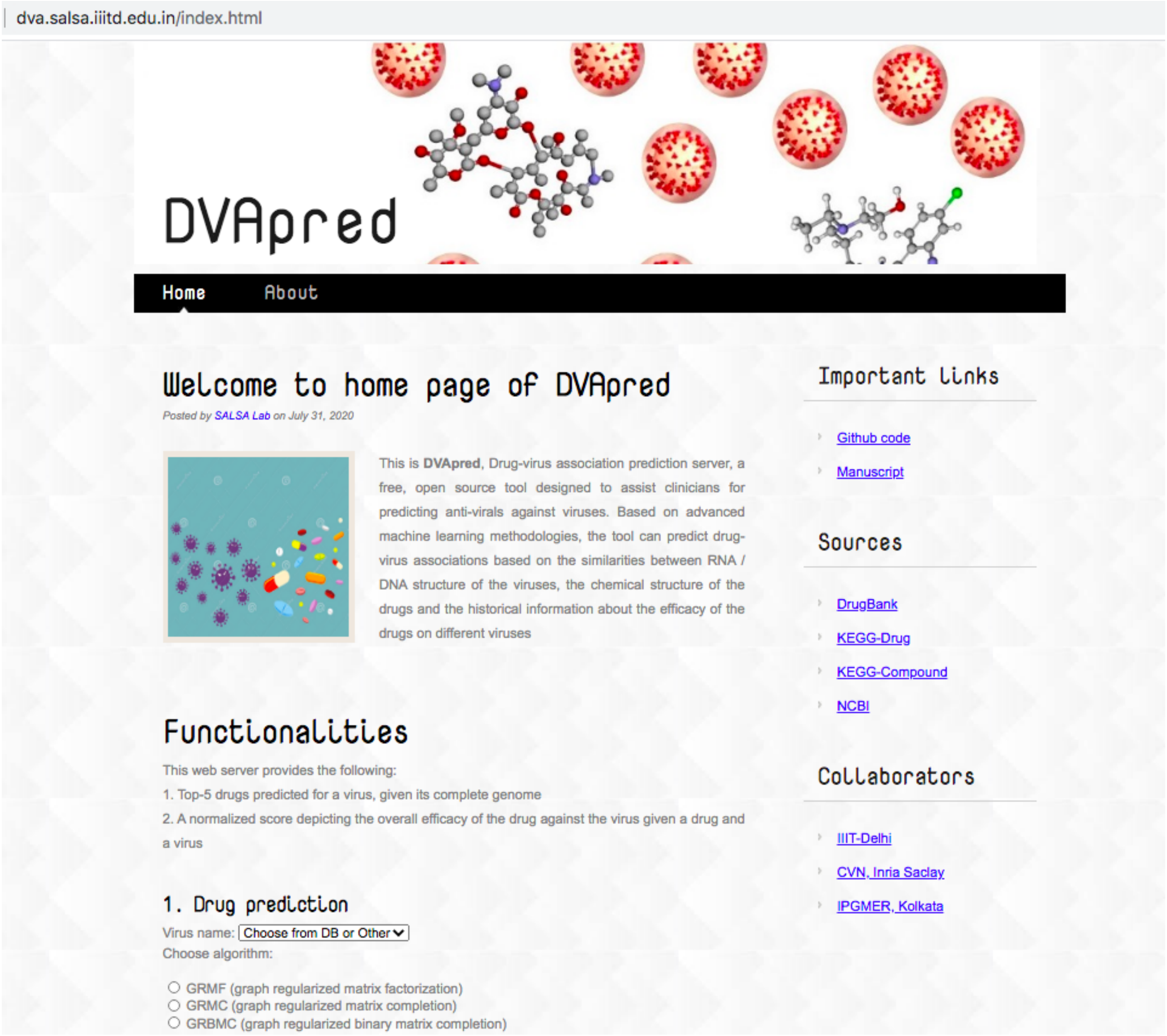

Figure 1: Home page of the web-server

About – This page contains of a brief description about how the dataset has been curated. It also gives short descriptions of the different algorithms used. The reference papers of the algorithms are also provided for interested

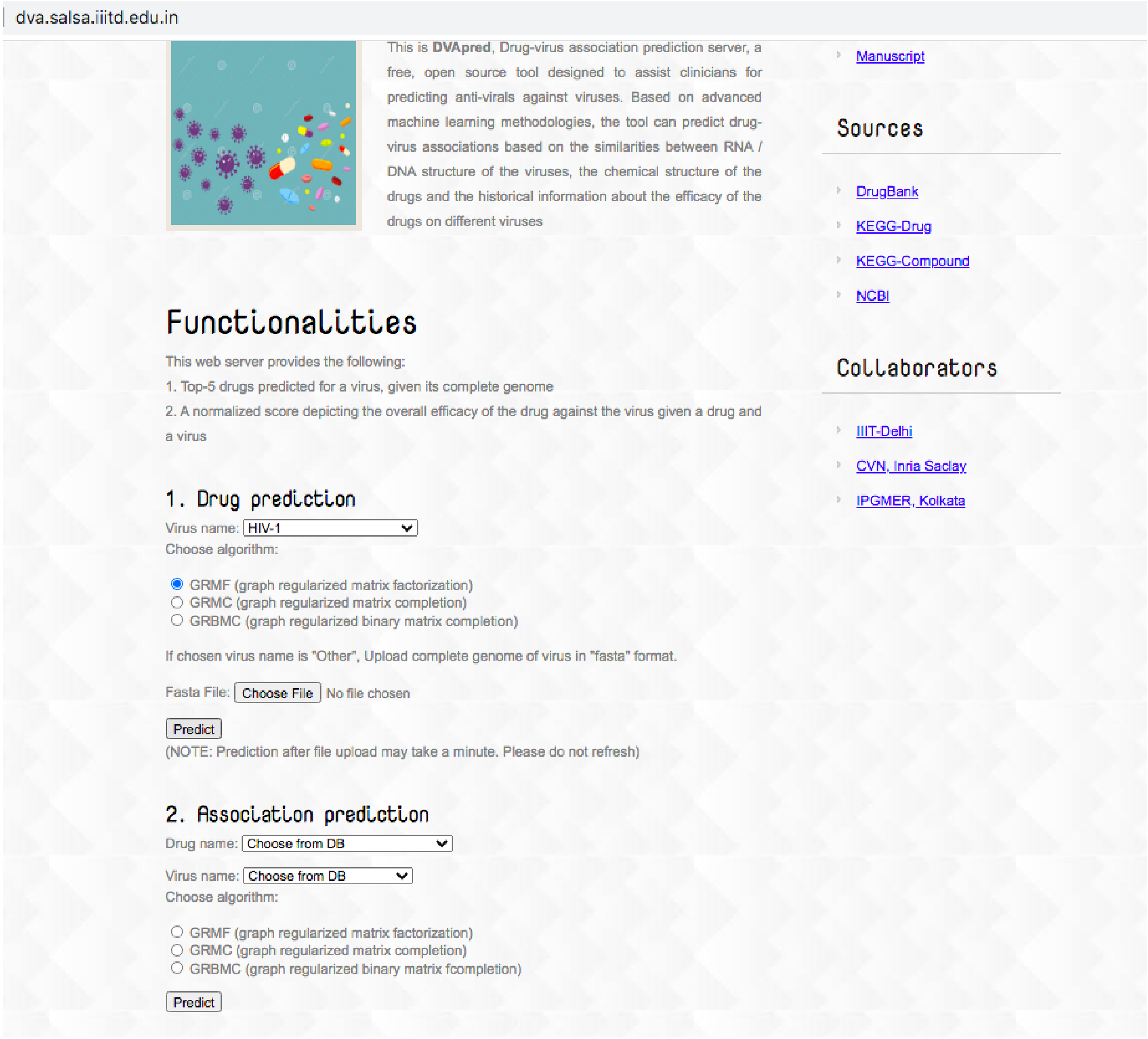

Figure 2: Sample query 1- To predict top-5 drugs for a virus already existing in the database

readers.

In the side menu, we also provide some important links, to our code on Github repository, our pre-print and other information pertaining to this work such as data sources and collaborations.

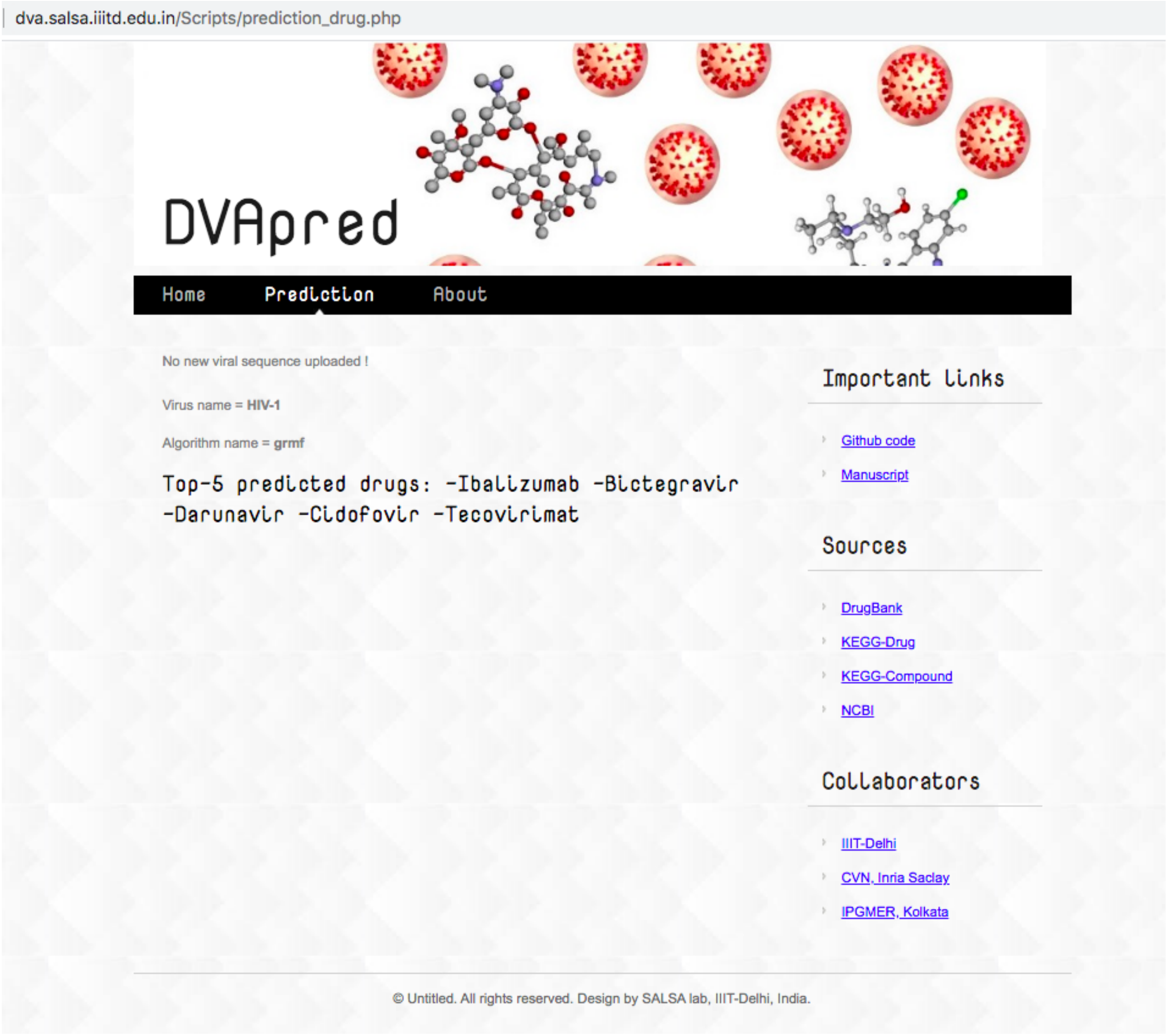

Figure 3: Sample query 1- Prediction results

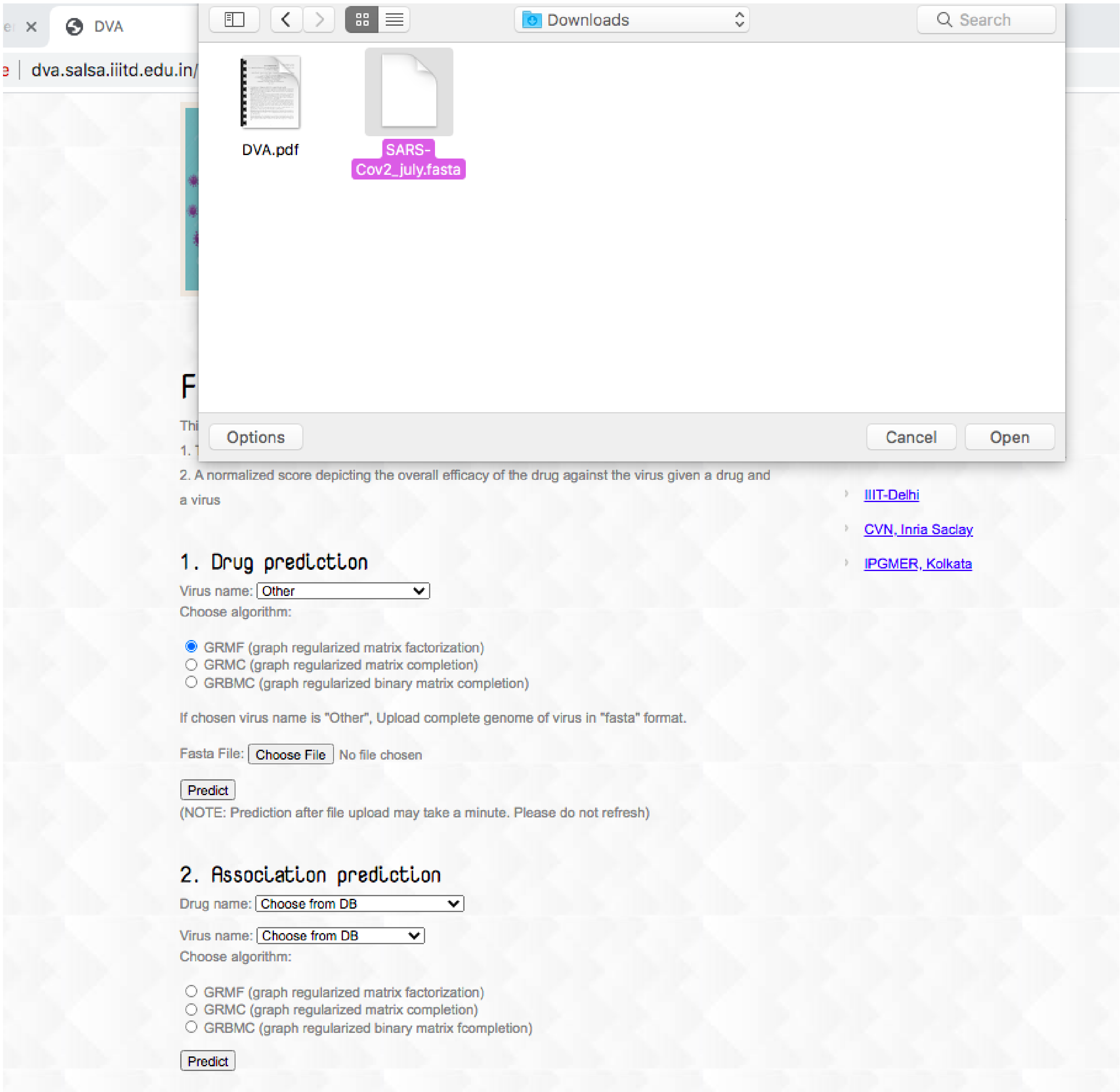

Figure 4: Sample query 2- To predict top-5 drugs for a novel virus by uploading its genomic sequence

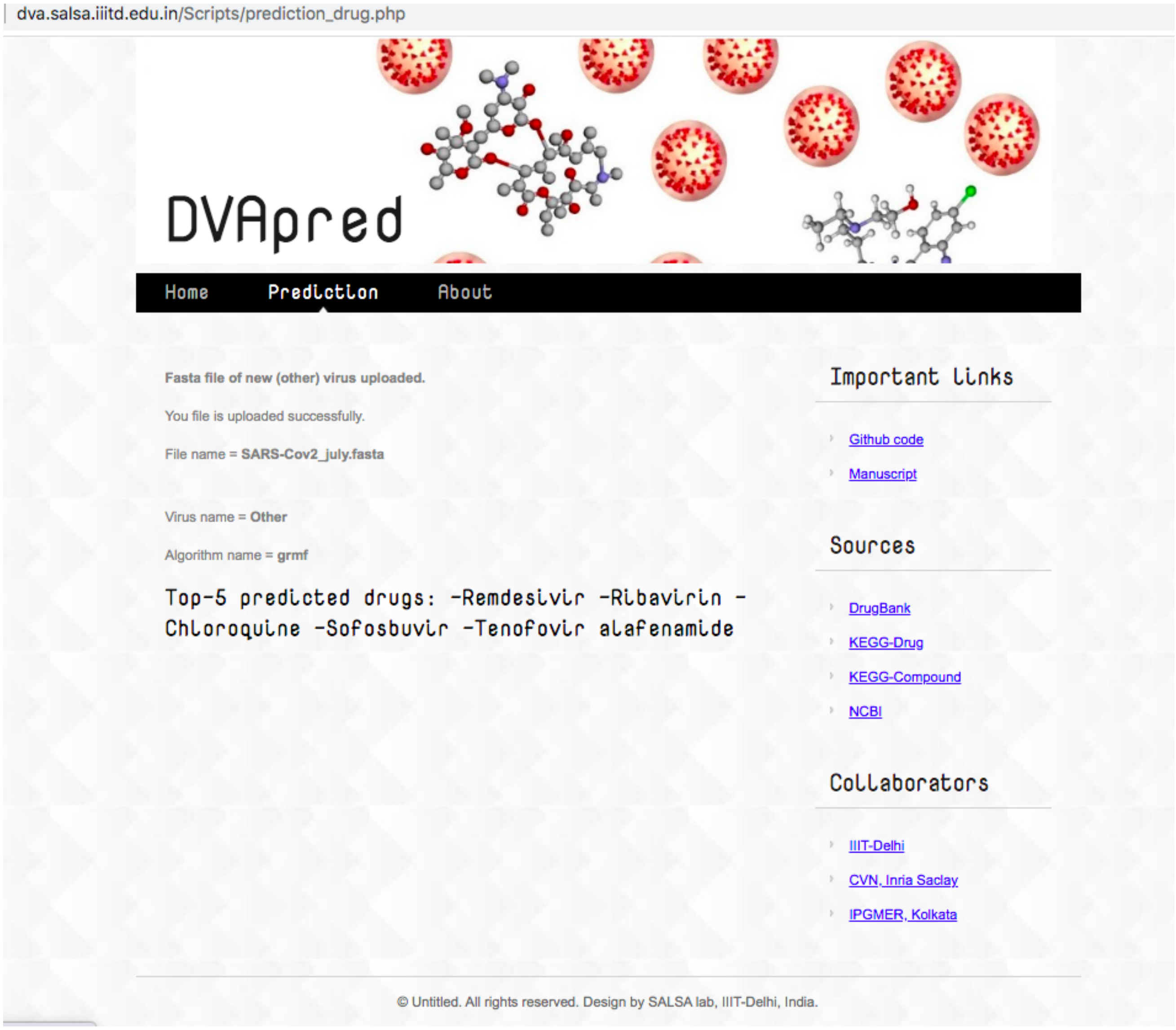

Figure 5: Sample query 2- Prediction results

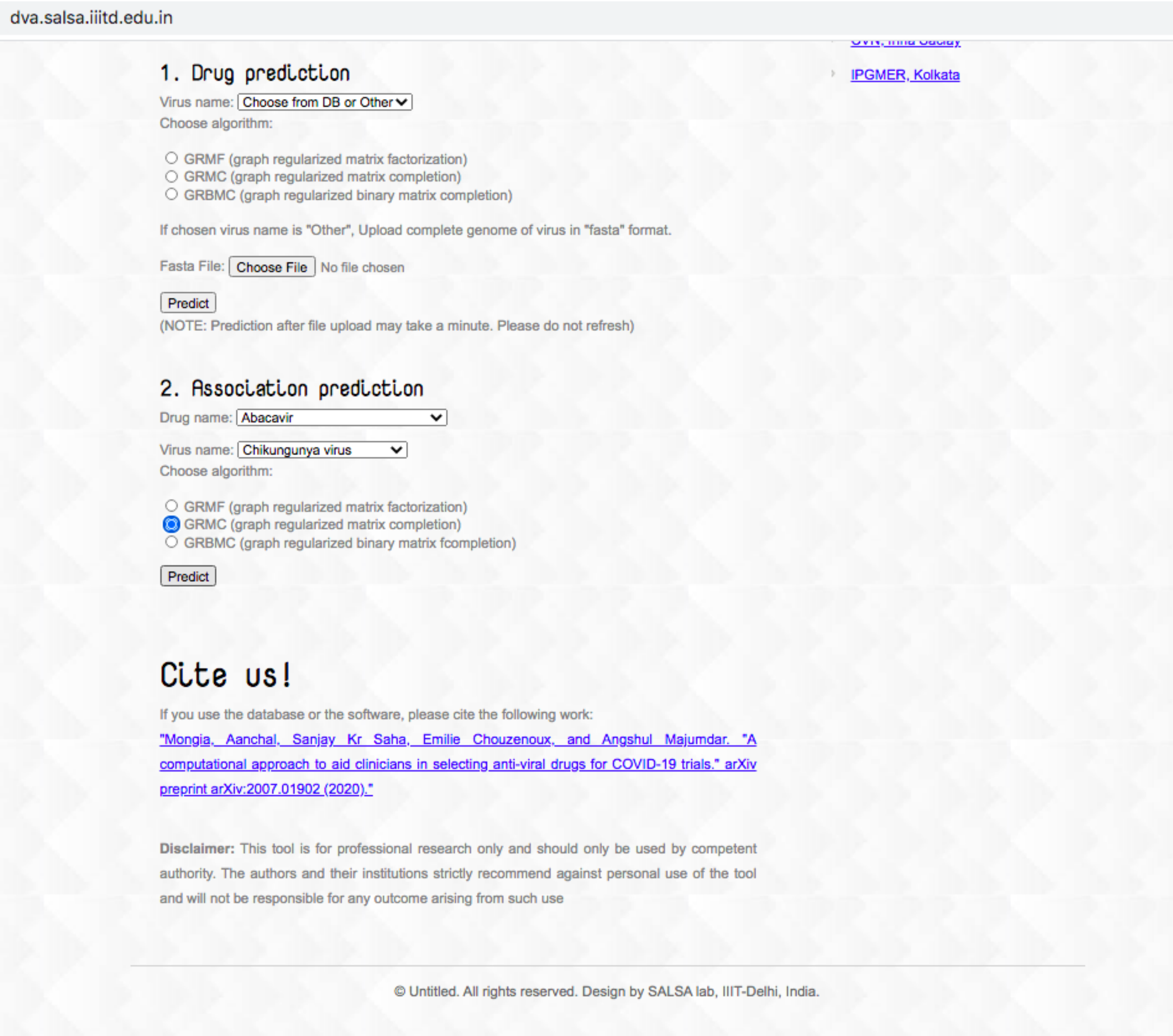

Figure 6: Sample query 3- To predict association score between a drug and virus.

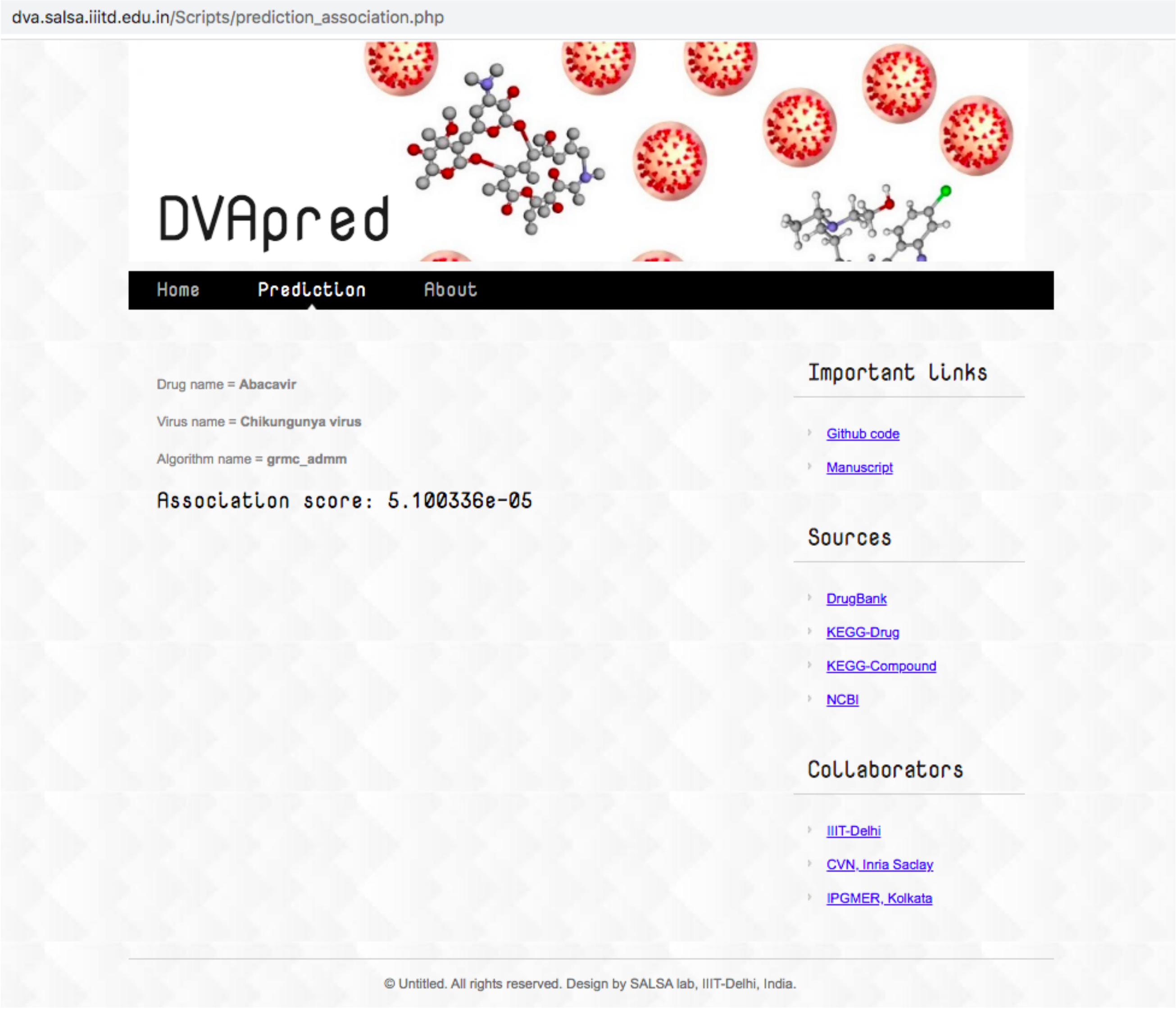

Figure 7: Sample query 3- Prediction results

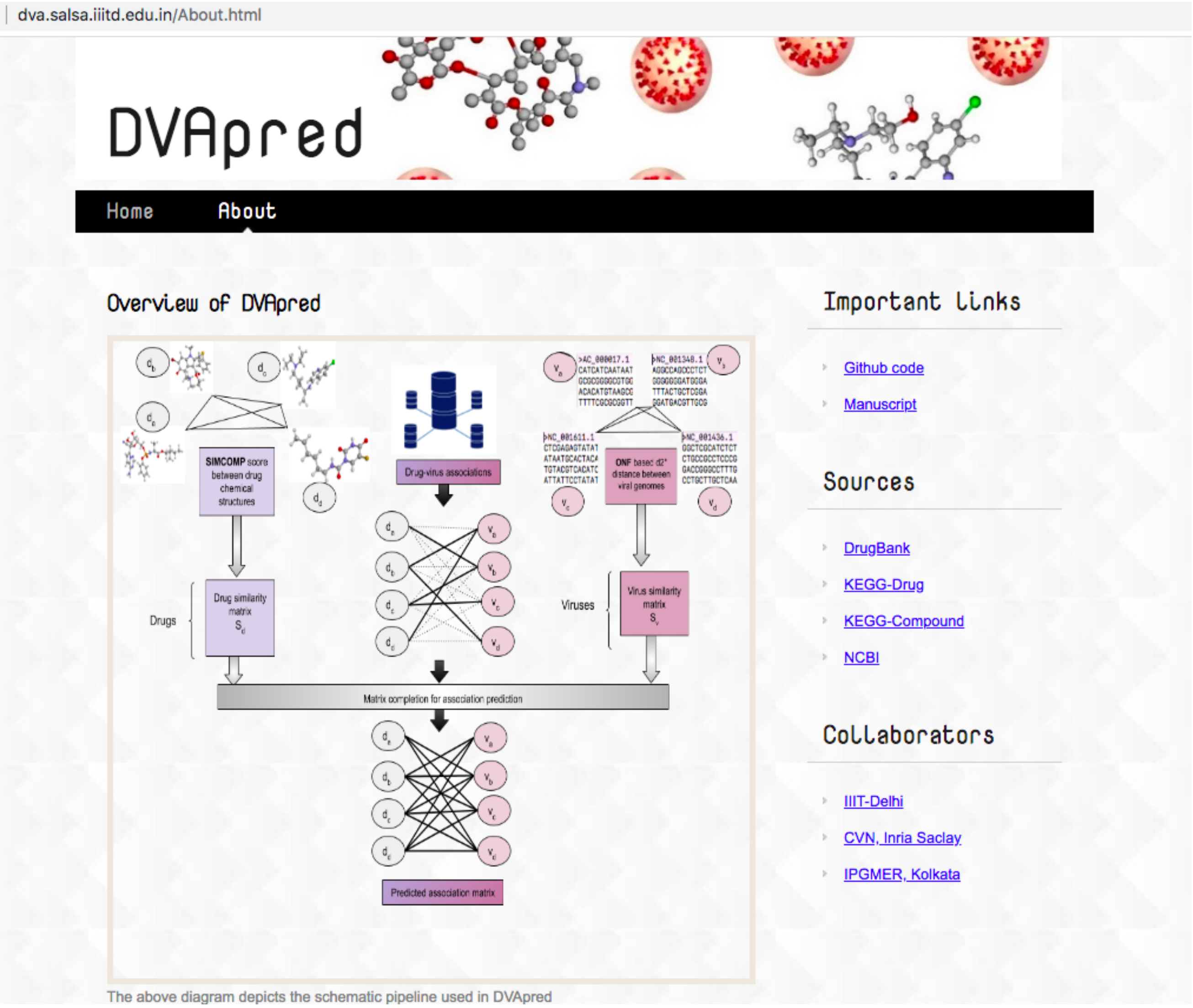

Figure 8: About page of the web-server
